# Use of intravitreal bevacizumab in a patient with a Von Hippel-Lindau-associated retinal haemangioblastoma of the optic nerve head: a case report

**DOI:** 10.1186/1752-1947-2-182

**Published:** 2008-05-29

**Authors:** Timothy A de Klerk, David HW Steel

**Affiliations:** 1 Sunderland Eye Infirmary, Queen Alexandra Road, Sunderland, Tyne and Wear SR2 9HP, UK

## Abstract

**Introduction:**

The optimum management of a capillary haemangioblastoma affecting the optic nerve head is not clear. A number of treatment modalities have been used to treat the tumours and their consequences. Ocular haemangioblastomas express high levels of vascular endothelial growth factor and levels have been correlated with tumour growth and activity. Treatment with vascular endothelial growth factor inhibitors would therefore seem a logical approach.

**Case presentation:**

We describe a 23-year-old man with an exophytic capillary haemangioblastoma of the optic nerve head that was treated with intravitreal bevacizumab injections.

**Conclusion:**

Unfortunately, treatment with intravitreal bevacizumab on three occasions had no effect on either tumour size or exudation in this patient.

## Introduction

The optimum management of capillary haemangioblastoma affecting the optic nerve head is not clear and indeed it has been described 'as one of the most difficult conditions to manage in ophthalmology' [1]. A number of treatment modalities have been used to treat the tumours and their consequences including argon laser photocoagulation, transpupillary thermotherapy, radiotherapy and vitrectomy surgery [2,3]. The tumours however are intrinsically related to the neurosensory retina and optic nerve and treatment often results in adjacent neural damage [2]. Ocular haemangioblastomas express high levels of vascular endothelial growth factor (VEGF) and levels have been correlated with tumour growth and activity [4]. Treatment with VEGF inhibitors would therefore seem a logical approach. A reduction in macular oedema and exudation has been described following systemic treatment with the intravenously delivered VEGF tyrosine kinase receptor inhibitor SU5416 [5,6]. We describe a patient with an exophytic capillary haemangioblastoma of the optic nerve head that was treated with intravitreal bevacizumab injections.

## Case presentation

**Figure 1:**
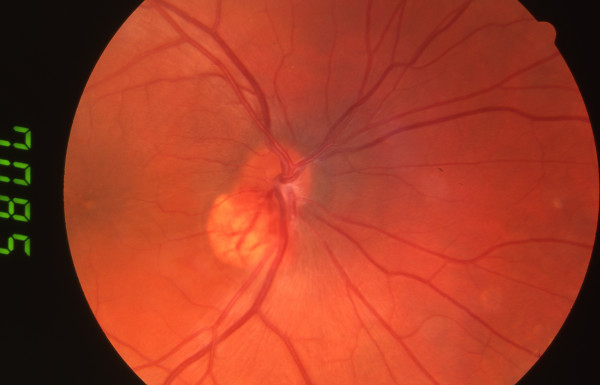
Fundal Photo – Two years after presentation showing an exophytic haemangioblastoma adjacent to the right optic nerve head.

**Figure 2:**
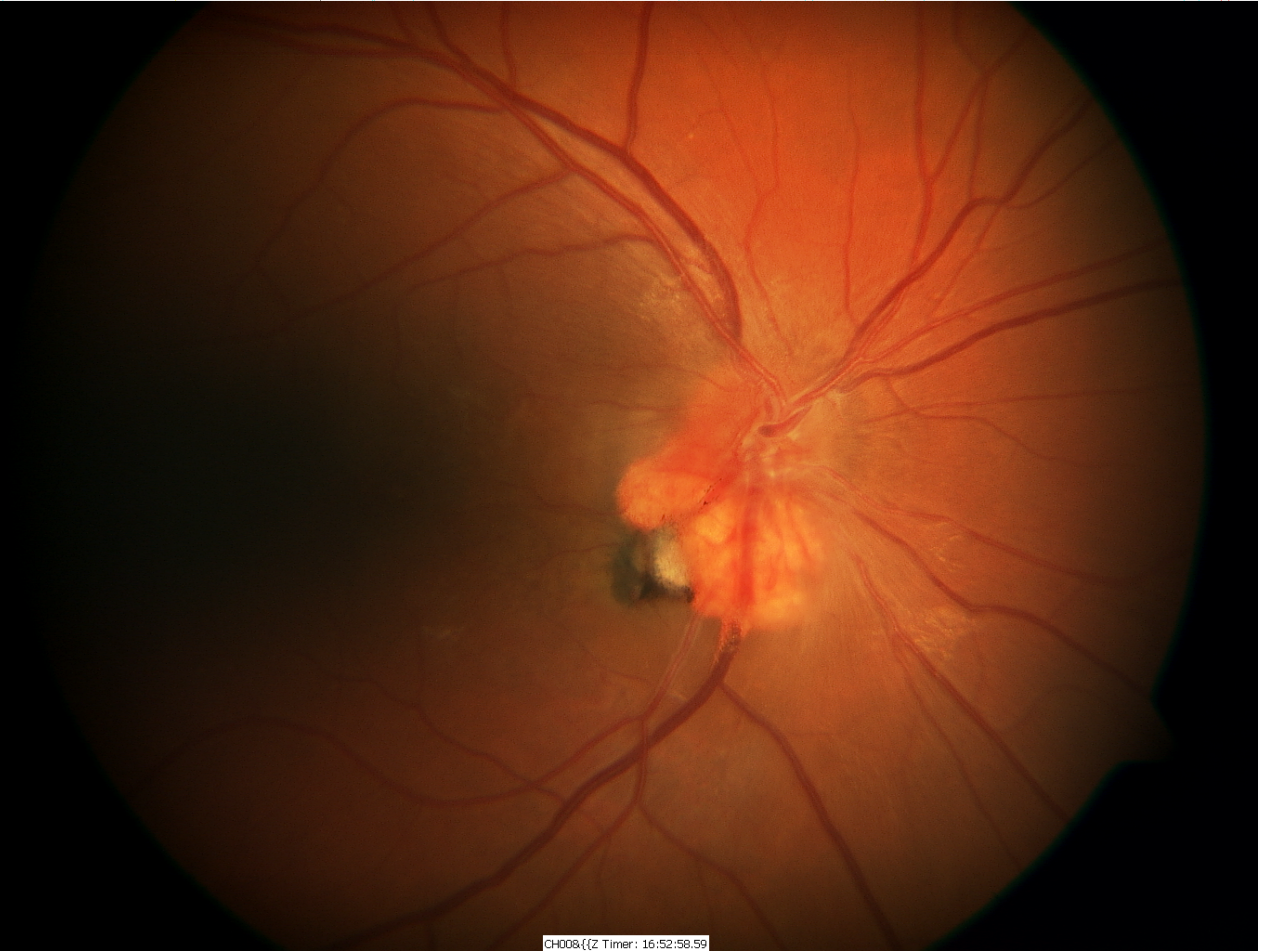
Fundal Photo – Six years after presentation, post argon laser therapy; note the pigmentation at the site of the laser.

**Figure 3:**
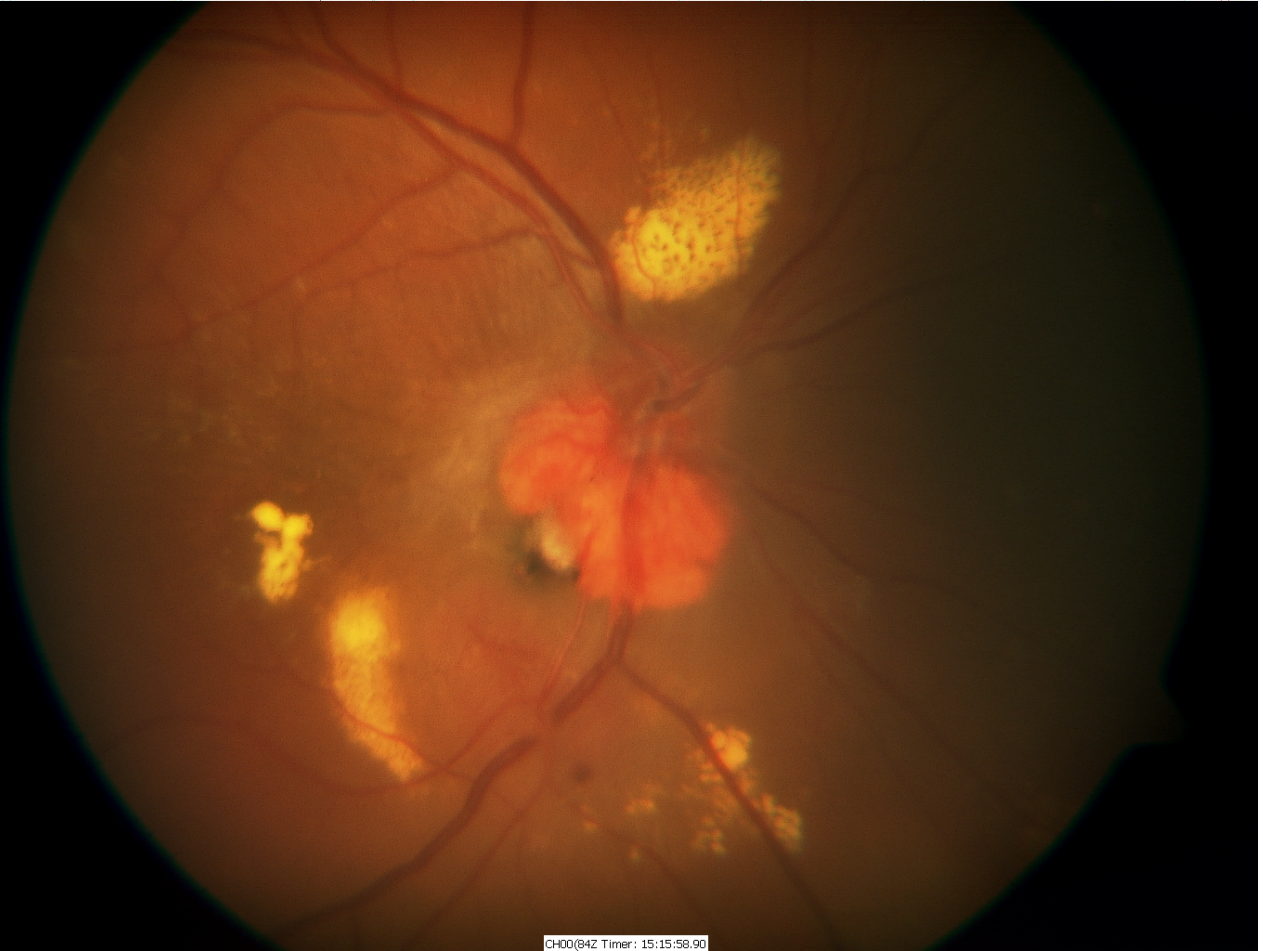
Fundal Photo – Thirteen years after presentation showing increasing exudation.

A 23-year-old man with Von Hippel-Lindau (VHL) disease developed a gradually enlarging exophytic haemangioblastoma adjacent to his right optic nerve head (Figure 1). After 5 years of followup he developed a serous detachment of his fovea and argon laser photocoagulation was carried out with direct treatment of the inferotemporal portion of the haemangioblastoma using low power (approximately 120 mW) long duration (0.5 seconds) burns. Treatment was carried out on five occasions at 3-month intervals resulting in a gradual reabsorption of the fluid but a reduction in visual acuity from 6/12 to 6/24 with a superonasal field defect (Figure 2). The patient was then observed with no further treatment being required until 7 years later when he again developed progressive exudation and serous peripapillary retinal detachment involving his fovea, reducing his visual acuity to 3/18 (Figure 3). This coincided with a progressive enlargement of three cerebellar haemangioblastomas, which were being observed without treatment. A number of treatment options were considered for his retinal lesion including further argon laser and transpupillary thermotherapy. However, because of previously reduced vision with laser photocoagulation the patient declined further laser therapy. Treatment with intravitreal bevacizumab was suggested as an alternative possibility. After a full discussion of this option and observation of gradually increasing exudation over an 18-month period, the patient had three intravitreal injections of bevacizumab 1.25 mg in 0.05 ml given at 1-month intervals. Refracted visual acuity, visual fields, colour photography, ultrasound and clinical examination with slit lamp biomicroscopy were carried out before, 1 and 3 months after the third intravitreal injection.

There was no improvement in any of the parameters measured. There was no reduction in tumour size on ultrasonography or clinically, and no reduction in exudates, macular oedema or area of serous detachment. Visual acuity continued to decline subjectively but remained objectively stable with a refracted acuity of 6/36 and n18 for near. Visual fields remained unchanged.

## Discussion

Treatment with intravitreal bevacizumab on three occasions had no effect on either tumour size or exudation in this patient with a capillary haemangioblastoma of the optic nerve head. Two previously documented cases treated with the systemic VEGF inhibitor SU5416 have reported a reduction in macular oedema and an improvement in visual acuity whilst undergoing treatment but a relapse following treatment withdrawal [5,6]. There was no change in tumour size despite treatment for 7 months in one of the cases [5]. There have been two other reports of intravitreal VEGF inhibitor treatment using pegaptanib for patients with juxtapapillary or large peripheral haemangioblastomas. In the first [7], two patients with optic disc haemangioblastomas received six to nine pegaptanib injections which resulted in an improvement in retinal exudation and, to a lesser extent, macular oedema but again no significant reduction in tumour size. Both patients had had prior vitrectomies, which may have altered the pharmacokinetics of the drug in the eye. In the second [8], two of the five patients enrolled in the study completed the full treatment course of intravitreal pegaptanib injections which comprised an injection every 6 weeks for a minimum of six injections. Lesions in the three who did not complete the course continued to progress, while retinal exudates and central retinal thickness decreased in the other two. Of the latter, one patient had an improvement in visual acuity of three lines; however, neither showed a reduction in leakage on fluorescein angiogram or tumour size.

We chose to use bevacizumab based on its ability to block all isoforms of VEGF and its availability. The lack of response we observed may be due to a number of possibilities.

Firstly, bevacizumab is a large molecule with potentially less effective retinal penetration than pegaptanib and therefore less effect on reducing vasopermeability of affected vessels and the tumour itself. Ranibizumab may offer superior efficacy, being a smaller molecule with better retinal penetration and having a 16-fold higher affinity for VEGF binding sites than bevacizumab [9]. Secondly, the intravitreally administered bevacizumab would have had little or no effect on systemically derived VEGF. The patient had three separate cerebellar haemangioblastomas, which may have been driving the intraocular process to some extent.

Thirdly, several hypoxia-induced genes are upregulated in VHL [4]. VHL is caused by a germline alteration of the VHL gene, which is a tumour suppressor gene. VHL mRNA encodes a protein termed pVHL and a lack of pVHL results in a lack of hypoxia inducible factor degradation, inducing profound intracellular changes that resemble the changes observed after oxidative stress. This results in increased levels of several factors including VEGF, erythropoietin, platelet-derived growth factor, and transforming growth factor as well as several other growth factors and cytokines. It is probably a combination of excessive amounts of these angiogenic factors that results in the formation of VHL-associated tumours. Blockage of more than just VEGF may therefore be needed to bring about tumour shrinkage or even reduced retinal exudation in some cases.

Finally, it is possible that, because the tumour and exudation had been present chronically, the three doses of bevacizumab used and the period of observation was too short to allow a clinically apparent improvement to occur. Further bevacizumab was not given because of the lack of any clinical benefit. Treatment at an earlier stage may have been more beneficial.

## Conclusion

In summary we observed no therapeutic response to three intravitreal bevacizumab injections in a patient with VHL and a peripapillary retinal haemangioblastoma. Treatment with ranibizumab in patients early in the course of their disease, who have no systemic tumours, could yield better results. Ultimately however, as Chan et al. have commented [4], a better therapeutic approach may be to target the VHL tumour cells directly or indeed the predisposing genetic defect.

## Abbreviations

VEGF: vascular endothelial growth factor; VHL: Von Hippel-Lindau

## Competing interests

The authors declare that they have no competing interests.

## Authors' contributions

TAD undertook background research and drafted the manuscript, DHWS conceived the idea, performed the procedure and drafted the manuscript. Both authors read and approved the final manuscript.

## Consent

Written informed consent was obtained from the patient for publication of this case report and accompanying images. A copy of the written consent is available for review by the Editor-in-Chief of this journal.
